# β-Cyclo­dextrin 10.41-hydrate

**DOI:** 10.1107/S160053680904865X

**Published:** 2009-11-21

**Authors:** Rüdiger W. Seidel, Bojidarka B. Koleva

**Affiliations:** a Lehrstuhl für Analytische Chemie, Ruhr-Universität Bochum, Universitätsstrasse 150, 44780 Bochum, Germany; b Department of Analytical Chemistry, University of Sofia St. Kliment Ohridski, J. Bourchier Blvd. 1, 1164 Sofia, Bulgaria

## Abstract

The crystal structure of β-cyclo­dextrin, C_42_H_70_O_35_·10.41H_2_O, consists of truncated cone-shaped β-cyclo­dextrin mol­ecules that are herringbone packed. The primary hydr­oxy groups form an intra­molecular hydrogen-bonded array. The semipolar cavity of the cyclo­dextrin host is filled with water mol­ecules, which show partial occupancy and disorder.

## Related literature

For an overview of cyclo­dextrin chemistry, see: Atwood *et al.* (1996 ▶), Szejtli (1998 ▶). For applications of cyclo­dextrins, see: Del Valle (2004 ▶). For previous X-ray crystal structure determinations of various β-cyclo­dextrin hydrates, see: Hamilton *et al.* (1968 ▶); Szejtli & Budai (1977 ▶); Lindner & Saenger (1978 ▶, 1982 ▶); Stezowski & Maclennan (1980 ▶); Fujiwara *et al.* (1983 ▶); Betzel *et al.* (1984 ▶); Steiner & Koellner (1994 ▶); Damodharan *et al.* (2004 ▶); Kurokawa, *et al.* (2004 ▶). For a low temperature single-crystal neutron diffraction study of deutero-β-CD·11D_2_O, see Zabel *et al.* (1986 ▶). For a description of the Cambridge Structural Database, see: Allen (2002 ▶).
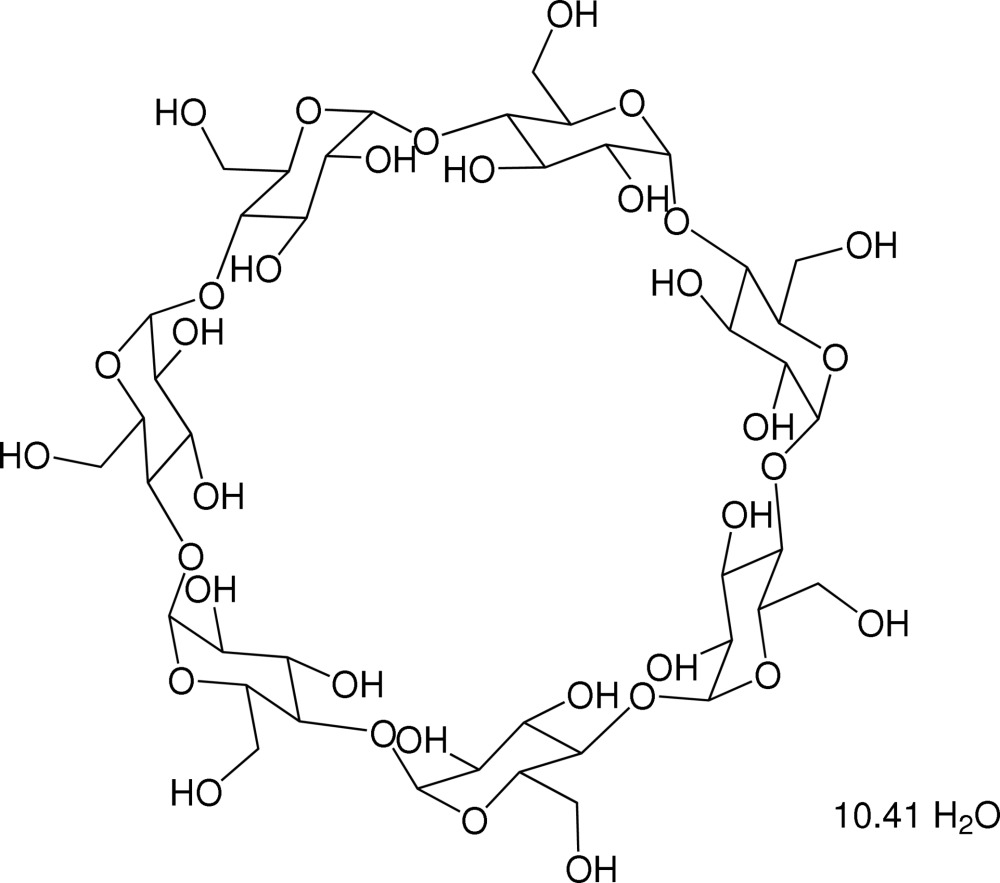


## Experimental

### 

#### Crystal data

C_42_H_70_O_35_·10.41H_2_O
*M* *_r_* = 1322.53
Monoclinic, 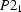

*a* = 20.8353 (4) Å
*b* = 9.9397 (1) Å
*c* = 15.2043 (3) Å
β = 110.630 (2)°
*V* = 2946.84 (9) Å^3^
*Z* = 2
Mo *K*α radiation
μ = 0.14 mm^−1^
*T* = 110 K
0.37 × 0.33 × 0.28 mm

#### Data collection

Oxford Diffraction Xcalibur^TM^2 diffractometer
Absorption correction: multi-scan (*ABSPACK* in *CrysAlis Pro*; Oxford Diffraction, 2009 ▶) *T*_min_ = 0.951, *T*_max_ = 0.963
40031 measured reflections
7019 independent reflections
6090 reflections with *I* > 2σ(*I*)
*R*_int_ = 0.026

#### Refinement

*R*[*F* ^2^ > 2σ(*F* ^2^)] = 0.032
*wR*(*F* ^2^) = 0.081
*S* = 0.99
7019 reflections
868 parameters
36 restraints
H atoms treated by a mixture of independent and constrained refinement
Δρ_max_ = 0.39 e Å^−3^
Δρ_min_ = −0.24 e Å^−3^

### 

Data collection: *CrysAlis Pro* (Oxford Diffraction, 2009 ▶); cell refinement: *CrysAlis Pro*; data reduction: *CrysAlis Pro*; method used to solve structure: initial coordinates of the β-cyclodextrin scaffold taken from Lindner & Saenger (1982 ▶); program(s) used to refine structure: *SHELXL97* (Sheldrick, 2008 ▶); molecular graphics: *DIAMOND* (Brandenburg, 2009 ▶); software used to prepare material for publication: *enCIFer* (Allen *et al.*, 2004 ▶).

## Supplementary Material

Crystal structure: contains datablocks global, I. DOI: 10.1107/S160053680904865X/tk2569sup1.cif

Structure factors: contains datablocks I. DOI: 10.1107/S160053680904865X/tk2569Isup2.hkl

Additional supplementary materials:  crystallographic information; 3D view; checkCIF report

## Figures and Tables

**Table 1:** Hydrogen-bond geometry (Å, °)

*D*—H⋯*A*	*D*—H	H⋯*A*	*D*⋯*A*	*D*—H⋯*A*
O1—H1*A*⋯O7	0.84	2.36	3.065 (3)	142
O2—H2*A*⋯O45^i^	0.84	2.00	2.809 (3)	161
O2—H2*A*⋯O45′^i^	0.84	1.95	2.633 (11)	138
O5—H5*A*⋯O47	0.84	1.76	2.578 (15)	163
O5—H5*A*⋯O45^ii^	0.84	2.46	3.024 (4)	125
O5′—H5′⋯O45^ii^	0.84	1.66	2.47 (3)	163
O5′—H5′⋯O45′^ii^	0.84	2.40	3.20 (3)	159
O6—H6*C*⋯O12	0.84	2.04	2.845 (2)	161
O6—H6*C*⋯O13	0.84	2.31	2.742 (2)	112
O7—H7*A*⋯O26^i^	0.84	2.14	2.956 (3)	165
O11—H11*A*⋯O40	0.84	1.78	2.595 (2)	163
O12—H12*C*⋯O31^iii^	0.84	1.85	2.676 (2)	167
O15—H15*A*⋯O41^iv^	0.84	2.04	2.870 (3)	172
O16—H16*A*⋯O38^v^	0.84	1.90	2.733 (3)	174
O17—H17*A*⋯O11	0.84	2.06	2.889 (2)	171
O20—H20*A*⋯O43	0.84	1.93	2.725 (3)	157
O20—H20*A*⋯O50	0.84	2.12	2.80 (3)	138
O21—H21*A*⋯O27	0.84	1.99	2.810 (2)	167
O21—H21*A*⋯O28	0.84	2.36	2.794 (2)	112
O22—H22*A*⋯O16	0.84	1.97	2.776 (2)	160
O22—H22*A*⋯O23	0.84	2.41	2.821 (2)	111
O25—H25*A*⋯O39^vi^	0.84	1.90	2.743 (3)	178
O26—H26*A*⋯O37^iii^	0.84	1.83	2.668 (3)	176
O27—H27*A*⋯O5^v^	0.84	2.09	2.821 (3)	145
O30—H30*C*⋯O15^vii^	0.84	2.06	2.858 (3)	158
O30—H30*C*⋯O14^vii^	0.84	2.37	2.945 (2)	126
O31—H31*A*⋯O2	0.84	2.01	2.844 (2)	172
O32—H32*A*⋯O26	0.84	2.04	2.871 (2)	170
O35—H35*A*⋯O37^i^	0.84	2.08	2.890 (2)	161
O36—H36⋯O11^ii^	0.84	2.02	2.854 (2)	173
O37—H37*A*⋯O6	0.824 (17)	2.05 (2)	2.837 (2)	160 (3)
O37—H37*B*⋯O32^viii^	0.849 (17)	1.910 (18)	2.753 (2)	171 (3)
O38—H38*A*⋯O20	0.828 (17)	2.11 (2)	2.865 (3)	152 (3)
O38—H38*B*⋯O30^ix^	0.817 (17)	1.857 (18)	2.672 (3)	176 (3)
O39—H39*A*⋯O22^ii^	0.856 (18)	1.898 (18)	2.749 (3)	173 (3)
O39—H39*B*⋯O38	0.861 (18)	2.18 (2)	2.959 (3)	151 (3)
O40—H40*A*⋯O41^x^	0.879 (18)	2.21 (3)	2.787 (3)	123 (3)
O40—H40*B*⋯O35^xi^	0.869 (18)	1.91 (2)	2.770 (3)	168 (3)
O41—H41*A*⋯O20	0.844 (17)	2.134 (18)	2.976 (3)	175 (3)
O41—H41*A*⋯O19	0.844 (17)	2.63 (3)	3.048 (2)	112 (3)
O41—H41*B*⋯O25	0.840 (18)	1.95 (2)	2.769 (3)	166 (3)
O42—H42*C*⋯O21^ii^	0.852 (18)	2.09 (2)	2.932 (3)	173 (3)
O42—H42*D*⋯O48	0.805 (18)	1.90 (4)	2.53 (3)	135 (3)
O42—H42*D*⋯O39	0.805 (18)	2.34 (3)	3.038 (3)	146 (3)

Symmetry codes: (i) 
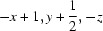
; (ii) 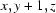; (iii) 
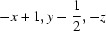
; (iv) 
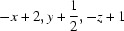
; (v) 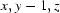; (vi) 
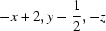
; (vii) 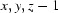; (viii) 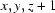; (ix) 
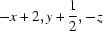
; (x) 
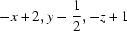
; (xi) 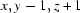.

**Fig. 1.**
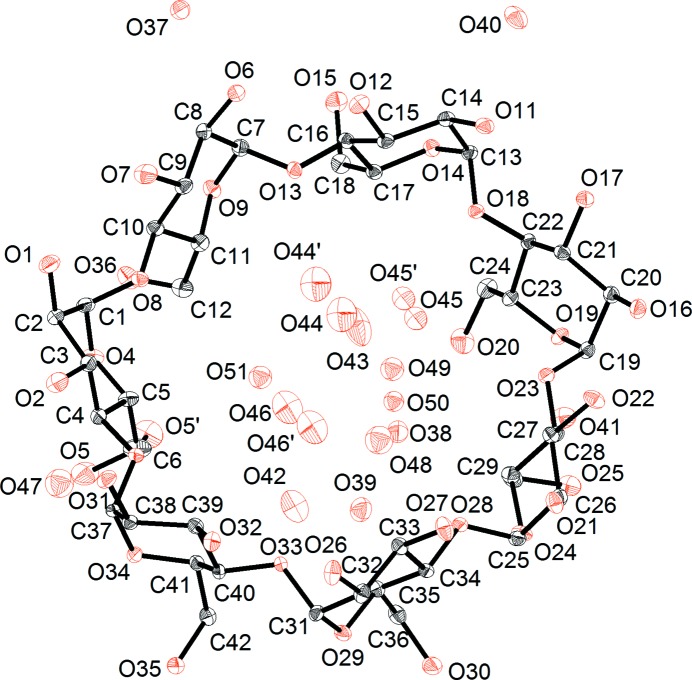
The asymmetric unit of (I), with displacement ellipsoids drawn at the 50% probability level. Positions of disordered O atoms with minor occupancy and those not fully occupied are drawn as empty ellipsoids. Hydrogen atoms are omitted for clarity.

**Fig. 2.**
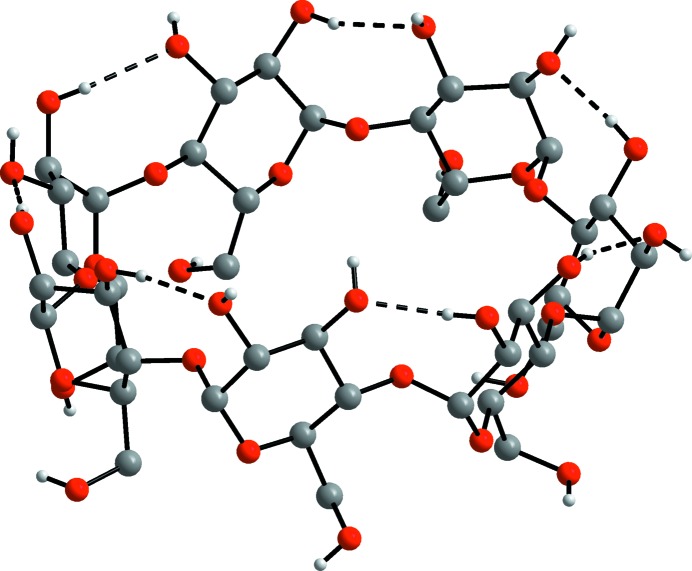
Molecular Structure of the β-cyclodextrin host in (I). H bonds are represented by dashed lines. H atoms attached to carbon are omitted for clarity.

**Fig. 3.**
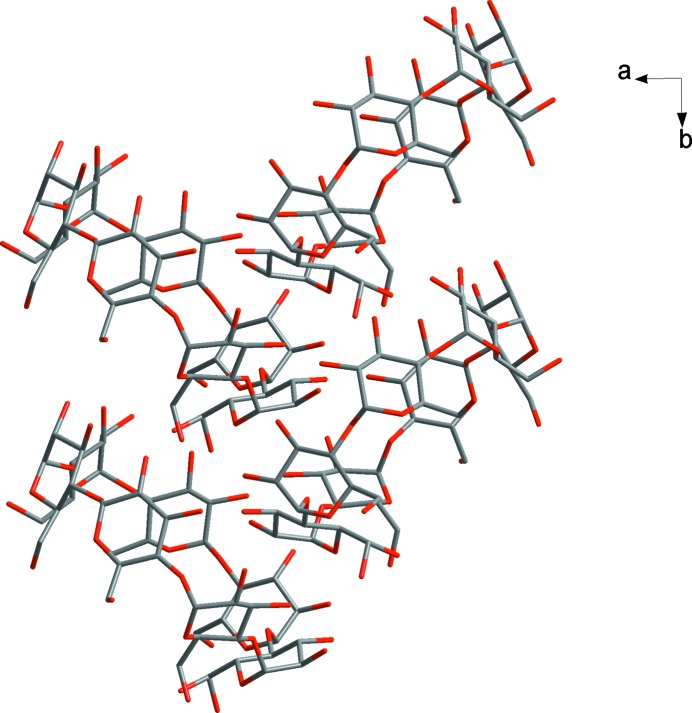
View of the crystal structure of (I) viewed down the c axis, showing the herringbone packing of the β-cyclodextrin molecules. H atoms and water molecules are omitted for clarity.

## supplementary crystallographic information

### Comment

Cyclodextrins are common, widely studied and cheaply available supramolecular hosts (Atwood *et al.*, 1996; Szejtli, 1998) with a variety of applications in the food, cosmetics and pharmaceutical industries (Del Valle, 2004).

β-Cyclodextrin (β-CD) is a cyclic oligosaccharide comprising seven *D*-glucopyranoside units, linked through 1,4-glycosidic bonds. The first room temperature crystal structure determination of β-CD dodecahydrate was reported about 40 years ago (Hamilton *et al.*, 1968). A number of room temperature determinations have been reported since (Szejtli & Budai, 1977; Lindner & Saenger, 1978; Lindner & Saenger, 1982; Fujiwara *et al.*, 1983; Betzel *et al.*, 1984; Steiner & Koellner, 1994; Damodharan *et al.*, 2004). A search of the Cambridge Structural Database (CSD; Version 5.3 with September 2009 Updates) (Allen *et al.*, 2002) revealed no low temperature X-ray structure determination of a β-CD hydrate with the exception of those reported by Stezowski & Maclennan (1980) and Kurokawa *et al.* (2004). Those were apparently reported without atomic coordinates and with an *R* factor of 13.0% and without refinement details, respectively. Additionally, a neutron diffraction study of deutero-β-CD 11 D_2_O at 120 K was reported by Zabel *et al.* (1986); the refinement of which converged at *R* = 0.049. Herein, we report an X-ray structural study of β-CD 10.41 hydrate at 110 K with *R* = 0.032 in order to provide an improved model of the β-CD host, (I).

Figs 1 and 2 show a displacement ellipsoid plot and an illustration of the molecular structure of the β-CD host, respectively. The shape of the molecule is a truncated cone. The geometric parameters lie within expected ranges. All *D*-glucopyranoside units exhibit the C1 chair conformation. The primary hydroxy groups at the wider end of the torus form an array of intramolecular H bonds with O—H···O contacts in the range of 2.776 (2)–3.065 (3) Å. The estimated volume of the semi-polar cavity of a β-CD host is 262 Å^3^ (Szejtli, 1998). In (I), the cavity was found to be statistically occupied by approximately 5.41 water molecules. Three of the water positions are each disordered over two positions, while five are considered to be partially occupied (see Refinement). In the vast number of structural studies dealing with β-CD hydrates, different solvent water contents were encountered. The solvent water content depends on the crystallization conditions and the humidity (Steiner & Koellner, 1994). In the crystal structure of (I), the β-CD molecules are arranged in herringbone-packed layers that are stacked along the *c* axis direction (Fig. 3).

### Experimental

Crystals of (I) were obtained unintentionally from an aqueous solution of β-cyclodextrin during an attempt to prepare an inclusion compound with an organic dye. A heterogeneous solid of (I) and the dye was obtained instead of the desired inclusion compound when the solvent was allowed to evaporate slowly. The solid was filtered off and dried over P_4_O_10_. A colourless crystal of (I) could be separated for the X-ray analysis.

### Refinement

In the absence of significant anomalous scattering effects, 5934 Friedel pairs were merged. Anisotropic displacement parameters were introduced for all non-hydrogen atoms with the exception of O47, O48, O49, O50 and O51, the positions of which are not fully occupied. One of the secondary hydroxy groups of the β-cyclodextrin host was found to be disordered over two positions. The ratio of occupancies of O5 and O5' was refined by means of a free variable and converged at 0.917 (5):0.083 (5). Standard similarity restraints on geometry and displacement parameters as well as rigid bond restraints were applied to the disordered group. The O44, O45 and O46 atoms of the solvent water molecules were each found to be disordered over two positions. The refinement of the occupancies by means of a free variable in each case yielded: 0.903 (5):0.097 (5), 0.803 (6):0.197 (6) and 0.884 (9):0.116 (9) for O44, O45 and O46, respectively. The parts of disordered water oxygen atoms were each refined with equivalent anisotropic displacement parameters. The site occupancy factors of O47, O48, O49, O50 and O51 were allowed to refine freely to yield 0.167 (9), 0.081 (8), 0.061 (8), 0.060 (8) and 0.039 (8), respectively. Four of the calculated intermolecular O···O distances (O43···O50 *ca* 2.01 Å, O44···O49 *ca* 1.73 Å, O45···O47 *ca* 2.29 Å and O46···O51 *ca* 2.42) indicate that the two positions cannot be occupied simultaneously in each case. The C-bound H atoms were placed at geometrically calculated positions (C—H = 0.99-1.00 Å) and refined with a riding model and with *U*_iso_(H) = 1.2 *U*_eq_(C). The hydroxy- and water-H atoms were localized in difference Fourier syntheses. The hydroxy-H atoms were subsequently refined with O—H = 0.84 Å and constrained tetrahedral C—O—H angles. The O—H bond lengths of the water molecules were restrained to a target value of 0.84 (2) Å. The 1,3-H,H distances of the water molecules were restrained to be similar with an effective standard deviation of 0.04 Å. The hydroxy- and water-H atoms were refined with *U*_iso_(H) = 1.2 *U*_eq_(O). The positions for some of the H atoms in some of the water molecules could not be determined reliably and were therefore excluded from the refinement.

### Crystal data

**Table:**

C_42_H_70_O_35_·10.41H_2_O	*F*(000) = 1412
*M**_r_* = 1322.53	*D*_x_ = 1.490 Mg m^−^^3^
Monoclinic, *P*2_1_	Mo *K*α radiation, λ = 0.71073 Å
Hall symbol: P 2yb	Cell parameters from 20832 reflections
*a* = 20.8353 (4) Å	θ = 2.7–28.1°
*b* = 9.9397 (1) Å	µ = 0.14 mm^−^^1^
*c* = 15.2043 (3) Å	*T* = 110 K
β = 110.630 (2)°	Prism, colourless
*V* = 2946.84 (9) Å^3^	0.37 × 0.33 × 0.28 mm
*Z* = 2	

### Data collection

**Table:**

Oxford Diffraction Xcalibur diffractometer	7019 independent reflections
Radiation source: Enhance (Mo) X-ray Source	6090 reflections with *I* > 2σ(*I*)
graphite	*R*_int_ = 0.026
Detector resolution: 8.4171 pixels mm^-1^	θ_max_ = 27.5°, θ_min_ = 2.9°
ω scans	*h* = −26→26
Absorption correction: multi-scan (ABSPACK in *CrysAlis PRO*; Oxford Diffraction, 2009)	*k* = −12→12
*T*_min_ = 0.951, *T*_max_ = 0.963	*l* = −19→19
40031 measured reflections	

### Refinement

**Table:**

Refinement on *F*^2^	Primary atom site location: the initial coordinates of the β-cyclodextrin scaffold were taken from Lindner & Saenger (1982)
Least-squares matrix: full	Secondary atom site location: difference Fourier map
*R*[*F*^2^ > 2σ(*F*^2^)] = 0.032	Hydrogen site location: inferred from neighbouring sites
*wR*(*F*^2^) = 0.081	H atoms treated by a mixture of independent and constrained refinement
*S* = 0.99	*w* = 1/[σ^2^(*F*_o_^2^) + (0.0578*P*)^2^] where *P* = (*F*_o_^2^ + 2*F*_c_^2^)/3
7019 reflections	(Δ/σ)_max_ = 0.001
868 parameters	Δρ_max_ = 0.39 e Å^−^^3^
36 restraints	Δρ_min_ = −0.24 e Å^−^^3^

### Special details

**Table:**

Geometry. All e.s.d.'s (except the e.s.d. in the dihedral angle between two l.s. planes) are estimated using the full covariance matrix. The cell e.s.d.'s are taken into account individually in the estimation of e.s.d.'s in distances, angles and torsion angles; correlations between e.s.d.'s in cell parameters are only used when they are defined by crystal symmetry. An approximate (isotropic) treatment of cell e.s.d.'s is used for estimating e.s.d.'s involving l.s. planes.
Refinement. Refinement of *F*^2^ against ALL reflections. The weighted *R*-factor *wR* and goodness of fit *S* are based on *F*^2^, conventional *R*-factors *R* are based on *F*, with *F* set to zero for negative *F*^2^. The threshold expression of *F*^2^ > σ(*F*^2^) is used only for calculating *R*-factors(gt) *etc*. and is not relevant to the choice of reflections for refinement. *R*-factors based on *F*^2^ are statistically about twice as large as those based on *F*, and *R*- factors based on ALL data will be even larger.

### Fractional atomic coordinates and isotropic or equivalent isotropic displacement parameters (Å^2^)

**Table:**

	*x*	*y*	*z*	*U*_iso_*/*U*_eq_	Occ. (<1)
O1	0.43109 (9)	0.4795 (2)	0.06964 (12)	0.0237 (4)	
H1A	0.4451	0.4043	0.0941	0.028*	
O2	0.41763 (9)	0.4374 (2)	−0.12954 (12)	0.0238 (4)	
H2A	0.3885	0.3942	−0.1142	0.029*	
O3	0.54177 (8)	0.39543 (17)	−0.15508 (11)	0.0154 (3)	
O4	0.59435 (9)	0.59993 (18)	0.05896 (11)	0.0207 (4)	
O5	0.64406 (13)	0.6978 (2)	−0.07616 (17)	0.0368 (7)	0.917 (5)
H5A	0.6758	0.7505	−0.0465	0.044*	0.917 (5)
C6	0.65945 (15)	0.5684 (3)	−0.03927 (19)	0.0294 (6)	0.917 (5)
H6A	0.7026	0.5711	0.0158	0.035*	0.917 (5)
H6B	0.6669	0.5091	−0.0871	0.035*	0.917 (5)
O5'	0.7150 (11)	0.620 (3)	0.0298 (15)	0.040 (5)	0.083 (5)
H5'	0.7025	0.6822	0.0573	0.059*	0.083 (5)
C6'	0.65945 (15)	0.5684 (3)	−0.03927 (19)	0.0294 (6)	0.083 (5)
H6A'	0.6756	0.4974	−0.0722	0.035*	0.083 (5)
H6B'	0.6391	0.6408	−0.0854	0.035*	0.083 (5)
O6	0.58391 (8)	0.16416 (19)	0.41646 (12)	0.0189 (4)	
H6C	0.6091	0.1016	0.4102	0.023*	
O7	0.49901 (9)	0.3003 (2)	0.24149 (13)	0.0260 (4)	
H7A	0.4845	0.3179	0.2853	0.031*	
O8	0.57078 (8)	0.42611 (17)	0.14463 (11)	0.0157 (3)	
O9	0.70256 (8)	0.41357 (17)	0.38138 (11)	0.0166 (4)	
O11	0.77159 (9)	−0.27659 (17)	0.45930 (12)	0.0177 (4)	
H11A	0.7667	−0.3267	0.5010	0.021*	
O12	0.66776 (8)	−0.06812 (18)	0.43729 (12)	0.0178 (4)	
H12C	0.6448	−0.1177	0.3926	0.021*	
O13	0.70736 (8)	0.18126 (17)	0.39046 (11)	0.0146 (3)	
O14	0.87332 (8)	0.03010 (17)	0.53239 (11)	0.0152 (3)	
O15	0.84497 (9)	0.26449 (19)	0.60728 (12)	0.0232 (4)	
H15A	0.8597	0.3377	0.6346	0.028*	
O16	0.92260 (9)	−0.49406 (17)	0.26437 (12)	0.0186 (4)	
H16A	0.9550	−0.5406	0.2600	0.022*	
O17	0.87877 (8)	−0.40676 (18)	0.41115 (11)	0.0179 (4)	
H17A	0.8453	−0.3770	0.4238	0.022*	
O18	0.86032 (8)	−0.11803 (17)	0.40904 (11)	0.0139 (3)	
O19	0.99620 (8)	−0.15637 (17)	0.31140 (11)	0.0154 (3)	
O20	0.99809 (10)	0.12933 (19)	0.33502 (15)	0.0297 (4)	
H20A	0.9622	0.1707	0.3035	0.045*	
O21	0.79734 (9)	−0.43799 (18)	−0.12551 (11)	0.0195 (4)	
H21A	0.7657	−0.3839	−0.1532	0.023*	
O22	0.85197 (9)	−0.47175 (18)	0.07245 (12)	0.0198 (4)	
H22A	0.8666	−0.4645	0.1313	0.024*	
O23	0.91264 (8)	−0.24262 (17)	0.17883 (11)	0.0150 (3)	
O24	0.91996 (8)	−0.15919 (18)	−0.05281 (11)	0.0175 (4)	
O25	1.04195 (9)	−0.1142 (2)	0.11668 (12)	0.0235 (4)	
H25A	1.0516	−0.1317	0.0687	0.028*	
O26	0.57670 (8)	−0.12304 (18)	−0.36578 (12)	0.0212 (4)	
H26A	0.5657	−0.1960	−0.3951	0.025*	
O27	0.68202 (9)	−0.29122 (18)	−0.23675 (13)	0.0230 (4)	
H27A	0.6554	−0.3069	−0.2073	0.035*	
O28	0.80158 (8)	−0.15711 (17)	−0.12746 (11)	0.0147 (3)	
O29	0.73057 (8)	0.07842 (18)	−0.32427 (11)	0.0159 (3)	
O30	0.87316 (9)	0.0327 (2)	−0.27399 (13)	0.0238 (4)	
H30C	0.8677	0.0859	−0.3190	0.036 (9)*	
O31	0.42147 (8)	0.30039 (18)	−0.29183 (12)	0.0194 (4)	
H31A	0.4242	0.3384	−0.2413	0.023*	
O32	0.48828 (8)	0.09599 (17)	−0.36044 (11)	0.0174 (3)	
H32A	0.5137	0.0286	−0.3554	0.021*	
O33	0.63304 (8)	0.11283 (16)	−0.28602 (11)	0.0137 (3)	
O34	0.58413 (8)	0.46343 (16)	−0.27119 (11)	0.0153 (3)	
O35	0.65236 (8)	0.44776 (18)	−0.39480 (11)	0.0178 (4)	
H35A	0.6216	0.5041	−0.3976	0.021*	
O36	0.70072 (9)	0.65475 (19)	0.26713 (13)	0.0251 (4)	
H36	0.7188	0.6715	0.3248	0.030*	
C1	0.54900 (12)	0.5510 (3)	0.10087 (16)	0.0164 (5)	
H1	0.5462	0.6171	0.1489	0.020*	
C2	0.47733 (12)	0.5308 (3)	0.02720 (16)	0.0170 (5)	
H2	0.4599	0.6208	−0.0002	0.020*	
C3	0.48101 (11)	0.4415 (3)	−0.05196 (15)	0.0163 (5)	
H3	0.4935	0.3481	−0.0274	0.020*	
C4	0.53467 (12)	0.4933 (2)	−0.08968 (16)	0.0162 (5)	
H4	0.5197	0.5815	−0.1221	0.019*	
C5	0.60351 (12)	0.5095 (3)	−0.00976 (16)	0.0181 (5)	
H5	0.6187	0.4199	0.0202	0.022*	
C7	0.68164 (12)	0.2994 (2)	0.41849 (16)	0.0161 (5)	
H7	0.6998	0.3052	0.4886	0.019*	
C8	0.60300 (12)	0.2877 (3)	0.38360 (16)	0.0159 (5)	
H8	0.5850	0.3637	0.4113	0.019*	
C9	0.57234 (12)	0.2998 (3)	0.27719 (16)	0.0160 (5)	
H9	0.5878	0.2200	0.2499	0.019*	
C10	0.60044 (12)	0.4254 (3)	0.24549 (15)	0.0157 (5)	
H10	0.5866	0.5080	0.2719	0.019*	
C11	0.67885 (12)	0.4133 (3)	0.28019 (16)	0.0164 (5)	
H11	0.6908	0.3246	0.2590	0.020*	
C12	0.71698 (13)	0.5221 (3)	0.24961 (18)	0.0209 (5)	
H12A	0.7668	0.5079	0.2822	0.025*	
H12B	0.7073	0.5122	0.1813	0.025*	
C13	0.85802 (12)	−0.1031 (2)	0.49974 (16)	0.0142 (5)	
H13	0.8922	−0.1654	0.5436	0.017*	
C14	0.78611 (12)	−0.1431 (2)	0.49470 (16)	0.0151 (5)	
H14	0.7839	−0.1397	0.5593	0.018*	
C15	0.73386 (11)	−0.0454 (2)	0.43193 (16)	0.0142 (5)	
H15	0.7313	−0.0574	0.3655	0.017*	
C16	0.75377 (11)	0.0987 (2)	0.46217 (16)	0.0140 (5)	
H16	0.7475	0.1162	0.5234	0.017*	
C17	0.82787 (12)	0.1293 (2)	0.47187 (16)	0.0142 (5)	
H17	0.8322	0.1264	0.4084	0.017*	
C18	0.85261 (13)	0.2635 (3)	0.51694 (17)	0.0188 (5)	
H18A	0.9013	0.2770	0.5244	0.023*	
H18B	0.8253	0.3369	0.4772	0.023*	
C19	0.97083 (12)	−0.2731 (2)	0.25883 (16)	0.0146 (5)	
H19	1.0073	−0.3129	0.2382	0.018*	
C20	0.94960 (12)	−0.3762 (2)	0.31866 (16)	0.0152 (5)	
H20	0.9903	−0.4010	0.3749	0.018*	
C21	0.89510 (11)	−0.3156 (2)	0.35013 (15)	0.0138 (5)	
H21	0.8530	−0.3000	0.2936	0.017*	
C22	0.91912 (11)	−0.1811 (2)	0.39884 (15)	0.0129 (5)	
H22	0.9551	−0.1961	0.4620	0.016*	
C23	0.94635 (11)	−0.0876 (2)	0.34095 (16)	0.0139 (5)	
H23	0.9074	−0.0573	0.2842	0.017*	
C24	0.98264 (13)	0.0341 (3)	0.39539 (18)	0.0198 (5)	
H24A	0.9532	0.0773	0.4261	0.024*	
H24B	1.0257	0.0054	0.4451	0.024*	
C25	0.86255 (12)	−0.2313 (2)	−0.11112 (16)	0.0152 (5)	
H25	0.8683	−0.2489	−0.1727	0.018*	
C26	0.85543 (12)	−0.3653 (2)	−0.06681 (16)	0.0162 (5)	
H26	0.8971	−0.4200	−0.0604	0.019*	
C27	0.85369 (12)	−0.3433 (2)	0.03141 (16)	0.0150 (5)	
H27	0.8115	−0.2916	0.0270	0.018*	
C28	0.91664 (12)	−0.2633 (2)	0.08818 (16)	0.0143 (5)	
H28	0.9589	−0.3157	0.0942	0.017*	
C29	0.91743 (12)	−0.1305 (3)	0.03929 (16)	0.0166 (5)	
H29	0.8745	−0.0795	0.0321	0.020*	
C30	0.97906 (12)	−0.0434 (3)	0.08994 (18)	0.0206 (5)	
H30A	0.9807	0.0323	0.0485	0.025*	
H30B	0.9734	−0.0050	0.1468	0.025*	
C31	0.66024 (11)	0.0500 (2)	−0.34856 (16)	0.0147 (5)	
H31	0.6349	0.0818	−0.4141	0.018*	
C32	0.64881 (12)	−0.1005 (3)	−0.34219 (16)	0.0163 (5)	
H32	0.6648	−0.1489	−0.3883	0.020*	
C33	0.68954 (12)	−0.1496 (2)	−0.24365 (16)	0.0156 (5)	
H33	0.6725	−0.1033	−0.1977	0.019*	
C34	0.76454 (12)	−0.1134 (2)	−0.22174 (15)	0.0135 (5)	
H34	0.7824	−0.1603	−0.2666	0.016*	
C35	0.77121 (11)	0.0386 (2)	−0.22966 (16)	0.0148 (5)	
H35	0.7525	0.0837	−0.1850	0.018*	
C36	0.84314 (12)	0.0888 (3)	−0.21093 (17)	0.0185 (5)	
H36A	0.8422	0.1881	−0.2169	0.022*	
H36B	0.8721	0.0659	−0.1456	0.022*	
C37	0.52490 (12)	0.4371 (2)	−0.24926 (15)	0.0144 (5)	
H37	0.4964	0.5206	−0.2598	0.017*	
C38	0.48255 (11)	0.3247 (2)	−0.31085 (16)	0.0145 (5)	
H38	0.4692	0.3534	−0.3780	0.017*	
C39	0.52559 (12)	0.1989 (2)	−0.29771 (16)	0.0139 (5)	
H39	0.5379	0.1666	−0.2315	0.017*	
C40	0.59077 (11)	0.2293 (2)	−0.31679 (16)	0.0125 (4)	
H40	0.5799	0.2433	−0.3856	0.015*	
C41	0.62916 (12)	0.3508 (2)	−0.26224 (16)	0.0138 (5)	
H41	0.6515	0.3266	−0.1944	0.017*	
C42	0.68285 (12)	0.3999 (2)	−0.30030 (16)	0.0157 (5)	
H42A	0.7096	0.4734	−0.2599	0.019*	
H42B	0.7149	0.3255	−0.2986	0.019*	
O37	0.45907 (9)	0.13938 (19)	0.45062 (12)	0.0208 (4)	
H37A	0.4952 (11)	0.126 (3)	0.4413 (19)	0.025*	
H37B	0.4700 (14)	0.134 (3)	0.5099 (13)	0.025*	
O38	1.02376 (10)	0.3565 (2)	0.23620 (13)	0.0248 (4)	
H38A	1.0311 (15)	0.290 (2)	0.271 (2)	0.030*	
H38B	1.0561 (13)	0.409 (3)	0.250 (2)	0.030*	
O39	0.92858 (10)	0.3215 (2)	0.04040 (14)	0.0299 (4)	
H39A	0.9075 (15)	0.387 (3)	0.055 (2)	0.036*	
H39B	0.9646 (12)	0.311 (3)	0.0898 (17)	0.036*	
O40	0.75974 (10)	−0.3861 (2)	0.60776 (14)	0.0293 (4)	
H40A	0.7851 (14)	−0.456 (3)	0.607 (2)	0.035*	
H40B	0.7227 (11)	−0.433 (3)	0.599 (2)	0.035*	
O41	1.11226 (10)	0.0083 (2)	0.28708 (14)	0.0282 (4)	
H41A	1.0788 (13)	0.038 (3)	0.300 (2)	0.034*	
H41B	1.0977 (15)	−0.029 (3)	0.2341 (16)	0.034*	
O42	0.77658 (12)	0.2871 (2)	−0.07080 (17)	0.0400 (5)	
H42C	0.7787 (17)	0.367 (2)	−0.090 (2)	0.048*	
H42D	0.8137 (12)	0.263 (4)	−0.036 (2)	0.048*	
O43	0.87096 (13)	0.2433 (3)	0.2749 (2)	0.0585 (7)	
O44	0.74718 (14)	0.1038 (3)	0.1869 (2)	0.0523 (8)	0.903 (5)
O44'	0.7000 (14)	0.109 (3)	0.1997 (19)	0.0523 (8)	0.097 (5)
O45	0.70193 (13)	−0.1792 (3)	0.1178 (2)	0.0287 (7)	0.803 (6)
O45'	0.7106 (6)	−0.1310 (13)	0.1604 (9)	0.0287 (7)	0.197 (6)
O46	0.68237 (15)	0.1660 (4)	0.0002 (3)	0.0502 (11)	0.884 (9)
O46'	0.6883 (13)	0.103 (3)	−0.033 (2)	0.0502 (11)	0.116 (9)
O47	0.7268 (7)	0.8957 (15)	−0.0091 (10)	0.044 (5)*	0.167 (9)
O48	0.8452 (15)	0.143 (3)	0.068 (2)	0.043 (11)*	0.081 (8)
O49	0.7843 (16)	0.013 (3)	0.120 (2)	0.030 (12)*	0.062 (8)
O50	0.8898 (15)	0.167 (3)	0.164 (2)	0.022 (11)*	0.060 (8)
O51	0.744 (3)	0.337 (5)	0.111 (3)	0.029 (18)*	0.039 (8)

### Atomic displacement parameters (Å^2^)

**Table:**

	*U*^11^	*U*^22^	*U*^33^	*U*^12^	*U*^13^	*U*^23^
O1	0.0206 (9)	0.0320 (11)	0.0213 (9)	0.0035 (8)	0.0110 (8)	−0.0001 (8)
O2	0.0146 (8)	0.0375 (11)	0.0167 (9)	0.0022 (8)	0.0023 (7)	−0.0030 (8)
O3	0.0204 (8)	0.0150 (8)	0.0118 (7)	0.0038 (7)	0.0069 (6)	0.0017 (6)
O4	0.0256 (9)	0.0228 (9)	0.0144 (8)	−0.0058 (8)	0.0081 (7)	−0.0017 (7)
O5	0.0467 (15)	0.0275 (13)	0.0468 (15)	−0.0080 (11)	0.0296 (12)	0.0026 (11)
C6	0.0291 (15)	0.0368 (16)	0.0245 (14)	−0.0102 (13)	0.0123 (12)	−0.0028 (12)
O5'	0.027 (8)	0.047 (9)	0.041 (9)	−0.010 (8)	0.007 (7)	0.001 (8)
C6'	0.0291 (15)	0.0368 (16)	0.0245 (14)	−0.0102 (13)	0.0123 (12)	−0.0028 (12)
O6	0.0178 (8)	0.0206 (9)	0.0211 (9)	0.0032 (7)	0.0105 (7)	0.0046 (7)
O7	0.0140 (8)	0.0407 (11)	0.0233 (9)	0.0001 (8)	0.0064 (7)	0.0040 (8)
O8	0.0183 (8)	0.0170 (8)	0.0108 (8)	0.0007 (7)	0.0040 (6)	−0.0001 (7)
O9	0.0181 (8)	0.0151 (8)	0.0139 (8)	−0.0009 (7)	0.0022 (7)	−0.0002 (7)
O11	0.0233 (9)	0.0139 (8)	0.0186 (9)	−0.0024 (7)	0.0108 (7)	0.0014 (7)
O12	0.0117 (8)	0.0208 (9)	0.0222 (9)	−0.0050 (7)	0.0076 (7)	−0.0038 (7)
O13	0.0135 (8)	0.0162 (8)	0.0116 (7)	0.0030 (7)	0.0014 (6)	0.0004 (6)
O14	0.0124 (8)	0.0178 (8)	0.0135 (8)	0.0002 (7)	0.0022 (6)	0.0000 (7)
O15	0.0275 (10)	0.0203 (9)	0.0218 (9)	−0.0042 (8)	0.0087 (8)	−0.0072 (7)
O16	0.0202 (9)	0.0157 (8)	0.0190 (8)	0.0029 (7)	0.0059 (7)	−0.0017 (7)
O17	0.0179 (8)	0.0180 (9)	0.0205 (8)	0.0006 (7)	0.0099 (7)	0.0040 (7)
O18	0.0115 (8)	0.0187 (9)	0.0121 (7)	0.0017 (7)	0.0048 (6)	0.0017 (6)
O19	0.0112 (8)	0.0188 (8)	0.0176 (8)	−0.0009 (7)	0.0068 (6)	−0.0027 (7)
O20	0.0291 (10)	0.0207 (10)	0.0408 (11)	−0.0065 (8)	0.0140 (9)	0.0032 (8)
O21	0.0190 (9)	0.0173 (9)	0.0159 (8)	0.0014 (7)	−0.0016 (7)	−0.0001 (7)
O22	0.0260 (9)	0.0171 (9)	0.0135 (8)	−0.0046 (8)	0.0034 (7)	0.0003 (7)
O23	0.0124 (8)	0.0206 (9)	0.0113 (8)	0.0028 (7)	0.0032 (6)	−0.0001 (7)
O24	0.0137 (8)	0.0253 (9)	0.0125 (8)	−0.0029 (7)	0.0032 (6)	0.0035 (7)
O25	0.0164 (8)	0.0309 (11)	0.0211 (9)	−0.0048 (8)	0.0040 (7)	0.0014 (8)
O26	0.0126 (8)	0.0187 (9)	0.0267 (9)	−0.0005 (7)	0.0002 (7)	−0.0049 (7)
O27	0.0184 (9)	0.0159 (9)	0.0314 (10)	−0.0015 (7)	0.0047 (8)	0.0052 (8)
O28	0.0140 (8)	0.0190 (8)	0.0108 (7)	0.0036 (7)	0.0043 (6)	0.0025 (6)
O29	0.0122 (8)	0.0206 (9)	0.0151 (8)	0.0015 (7)	0.0048 (6)	0.0050 (7)
O30	0.0195 (9)	0.0308 (11)	0.0241 (9)	0.0047 (8)	0.0115 (7)	0.0090 (8)
O31	0.0135 (8)	0.0268 (10)	0.0194 (8)	0.0015 (7)	0.0075 (7)	−0.0013 (7)
O32	0.0146 (8)	0.0156 (8)	0.0216 (8)	−0.0011 (7)	0.0058 (7)	−0.0012 (7)
O33	0.0156 (8)	0.0132 (8)	0.0130 (8)	0.0046 (7)	0.0059 (6)	0.0029 (6)
O34	0.0174 (8)	0.0130 (8)	0.0177 (8)	0.0011 (7)	0.0091 (7)	0.0014 (7)
O35	0.0177 (8)	0.0200 (9)	0.0171 (8)	0.0030 (7)	0.0077 (7)	0.0030 (7)
O36	0.0263 (10)	0.0214 (10)	0.0235 (9)	−0.0020 (8)	0.0039 (8)	0.0031 (8)
C1	0.0205 (12)	0.0173 (12)	0.0136 (11)	−0.0005 (10)	0.0086 (9)	−0.0013 (9)
C2	0.0204 (12)	0.0170 (12)	0.0155 (11)	0.0029 (10)	0.0088 (10)	−0.0004 (9)
C3	0.0151 (11)	0.0195 (12)	0.0124 (11)	0.0032 (10)	0.0023 (9)	−0.0003 (9)
C4	0.0202 (12)	0.0149 (12)	0.0140 (11)	0.0035 (10)	0.0065 (9)	0.0003 (9)
C5	0.0198 (12)	0.0225 (13)	0.0134 (11)	−0.0017 (10)	0.0076 (10)	−0.0016 (10)
C7	0.0167 (11)	0.0175 (12)	0.0125 (11)	0.0009 (10)	0.0031 (9)	−0.0011 (9)
C8	0.0149 (11)	0.0175 (12)	0.0153 (11)	0.0032 (10)	0.0054 (9)	0.0018 (9)
C9	0.0123 (11)	0.0217 (12)	0.0142 (11)	0.0007 (10)	0.0049 (9)	−0.0013 (10)
C10	0.0154 (11)	0.0199 (12)	0.0108 (11)	0.0011 (10)	0.0033 (9)	−0.0008 (9)
C11	0.0148 (11)	0.0184 (12)	0.0147 (11)	−0.0011 (10)	0.0035 (9)	−0.0022 (9)
C12	0.0160 (12)	0.0268 (14)	0.0175 (12)	−0.0034 (11)	0.0032 (10)	0.0008 (10)
C13	0.0139 (11)	0.0178 (12)	0.0110 (10)	0.0023 (9)	0.0045 (9)	0.0003 (9)
C14	0.0166 (11)	0.0160 (11)	0.0144 (11)	−0.0012 (9)	0.0075 (9)	0.0000 (9)
C15	0.0104 (10)	0.0190 (12)	0.0139 (11)	−0.0009 (9)	0.0053 (9)	0.0007 (9)
C16	0.0119 (11)	0.0163 (12)	0.0130 (10)	0.0001 (9)	0.0036 (8)	0.0017 (9)
C17	0.0130 (11)	0.0169 (12)	0.0120 (10)	0.0005 (9)	0.0037 (9)	0.0012 (9)
C18	0.0182 (12)	0.0176 (12)	0.0194 (12)	−0.0037 (10)	0.0051 (10)	−0.0018 (10)
C19	0.0115 (11)	0.0177 (12)	0.0135 (11)	0.0015 (9)	0.0030 (9)	−0.0014 (9)
C20	0.0150 (11)	0.0159 (12)	0.0130 (11)	0.0016 (9)	0.0029 (9)	0.0000 (9)
C21	0.0121 (10)	0.0164 (12)	0.0123 (10)	−0.0004 (9)	0.0036 (9)	0.0019 (9)
C22	0.0093 (10)	0.0162 (12)	0.0127 (10)	0.0020 (9)	0.0030 (8)	0.0001 (9)
C23	0.0114 (10)	0.0157 (11)	0.0155 (11)	−0.0005 (9)	0.0058 (9)	−0.0011 (9)
C24	0.0168 (12)	0.0188 (12)	0.0252 (13)	−0.0043 (10)	0.0093 (10)	−0.0028 (10)
C25	0.0127 (11)	0.0206 (13)	0.0125 (11)	0.0006 (9)	0.0045 (9)	0.0015 (9)
C26	0.0160 (11)	0.0164 (12)	0.0144 (11)	0.0030 (9)	0.0031 (9)	−0.0005 (9)
C27	0.0157 (11)	0.0140 (11)	0.0148 (11)	−0.0001 (9)	0.0049 (9)	0.0007 (9)
C28	0.0137 (11)	0.0166 (11)	0.0129 (11)	0.0001 (9)	0.0049 (9)	−0.0015 (9)
C29	0.0157 (12)	0.0186 (12)	0.0144 (11)	−0.0010 (10)	0.0040 (9)	0.0000 (9)
C30	0.0198 (12)	0.0188 (13)	0.0212 (12)	−0.0037 (10)	0.0046 (10)	0.0033 (10)
C31	0.0124 (11)	0.0181 (12)	0.0135 (11)	0.0039 (9)	0.0045 (9)	0.0000 (9)
C32	0.0118 (11)	0.0179 (12)	0.0171 (11)	0.0010 (9)	0.0023 (9)	−0.0025 (10)
C33	0.0173 (12)	0.0120 (11)	0.0177 (11)	−0.0014 (9)	0.0065 (9)	0.0006 (9)
C34	0.0143 (11)	0.0163 (12)	0.0099 (10)	0.0020 (9)	0.0041 (8)	0.0015 (9)
C35	0.0139 (11)	0.0160 (12)	0.0137 (11)	0.0028 (9)	0.0039 (9)	0.0018 (9)
C36	0.0173 (12)	0.0161 (12)	0.0207 (12)	−0.0007 (10)	0.0051 (9)	0.0033 (10)
C37	0.0154 (11)	0.0177 (12)	0.0123 (10)	0.0048 (9)	0.0076 (9)	0.0036 (9)
C38	0.0107 (11)	0.0201 (12)	0.0135 (11)	0.0022 (9)	0.0053 (9)	0.0031 (9)
C39	0.0138 (11)	0.0143 (11)	0.0124 (10)	−0.0004 (9)	0.0033 (9)	0.0006 (9)
C40	0.0120 (11)	0.0121 (11)	0.0141 (11)	0.0028 (9)	0.0053 (9)	0.0025 (9)
C41	0.0153 (11)	0.0132 (11)	0.0118 (10)	0.0019 (9)	0.0035 (9)	0.0011 (9)
C42	0.0157 (11)	0.0137 (11)	0.0163 (11)	−0.0007 (9)	0.0039 (9)	−0.0001 (9)
O37	0.0183 (9)	0.0247 (10)	0.0182 (9)	0.0013 (8)	0.0051 (7)	−0.0002 (7)
O38	0.0228 (10)	0.0224 (10)	0.0311 (11)	−0.0006 (8)	0.0118 (8)	0.0028 (8)
O39	0.0258 (10)	0.0335 (12)	0.0287 (10)	0.0057 (9)	0.0074 (8)	−0.0002 (9)
O40	0.0235 (10)	0.0296 (11)	0.0358 (11)	−0.0020 (9)	0.0115 (9)	0.0132 (9)
O41	0.0213 (10)	0.0391 (12)	0.0237 (10)	−0.0036 (9)	0.0074 (8)	−0.0022 (9)
O42	0.0337 (12)	0.0314 (12)	0.0432 (13)	−0.0093 (10)	−0.0011 (10)	0.0006 (11)
O43	0.0398 (14)	0.0378 (14)	0.094 (2)	−0.0030 (11)	0.0194 (14)	0.0242 (14)
O44	0.0400 (16)	0.0510 (17)	0.0615 (18)	−0.0047 (14)	0.0123 (13)	−0.0022 (14)
O44'	0.0400 (16)	0.0510 (17)	0.0615 (18)	−0.0047 (14)	0.0123 (13)	−0.0022 (14)
O45	0.0278 (12)	0.0282 (16)	0.0290 (16)	−0.0001 (11)	0.0088 (12)	0.0024 (12)
O45'	0.0278 (12)	0.0282 (16)	0.0290 (16)	−0.0001 (11)	0.0088 (12)	0.0024 (12)
O46	0.0424 (15)	0.046 (2)	0.055 (2)	−0.0070 (15)	0.0087 (14)	0.0050 (17)
O46'	0.0424 (15)	0.046 (2)	0.055 (2)	−0.0070 (15)	0.0087 (14)	0.0050 (17)

### Geometric parameters (Å, °)

**Table:**

O1—C2	1.429 (3)	C8—H8	1.0000
O1—H1A	0.8400	C9—C10	1.527 (3)
O2—C3	1.428 (3)	C9—H9	1.0000
O2—H2A	0.8400	C10—C11	1.534 (3)
O3—C37	1.411 (3)	C10—H10	1.0000
O3—C4	1.436 (3)	C11—C12	1.509 (3)
O4—C1	1.400 (3)	C11—H11	1.0000
O4—C5	1.441 (3)	C12—H12A	0.9900
O5—C6	1.395 (4)	C12—H12B	0.9900
O5—H5A	0.8400	C13—C14	1.526 (3)
C6—C5	1.507 (4)	C13—H13	1.0000
C6—H6A	0.9900	C14—C15	1.519 (3)
C6—H6B	0.9900	C14—H14	1.0000
O5'—H5'	0.8400	C15—C16	1.517 (3)
O6—C8	1.433 (3)	C15—H15	1.0000
O6—H6C	0.8400	C16—C17	1.529 (3)
O7—C9	1.430 (3)	C16—H16	1.0000
O7—H7A	0.8400	C17—C18	1.506 (3)
O8—C1	1.406 (3)	C17—H17	1.0000
O8—C10	1.437 (3)	C18—H18A	0.9900
O9—C7	1.403 (3)	C18—H18B	0.9900
O9—C11	1.441 (3)	C19—C20	1.536 (3)
O11—C14	1.424 (3)	C19—H19	1.0000
O11—H11A	0.8400	C20—C21	1.505 (3)
O12—C15	1.426 (3)	C20—H20	1.0000
O12—H12C	0.8400	C21—C22	1.525 (3)
O13—C7	1.417 (3)	C21—H21	1.0000
O13—C16	1.433 (3)	C22—C23	1.520 (3)
O14—C13	1.410 (3)	C22—H22	1.0000
O14—C17	1.449 (3)	C23—C24	1.510 (3)
O15—C18	1.437 (3)	C23—H23	1.0000
O15—H15A	0.8400	C24—H24A	0.9900
O16—C20	1.429 (3)	C24—H24B	0.9900
O16—H16A	0.8400	C25—C26	1.523 (3)
O17—C21	1.421 (3)	C25—H25	1.0000
O17—H17A	0.8400	C26—C27	1.522 (3)
O18—C13	1.405 (3)	C26—H26	1.0000
O18—C22	1.432 (3)	C27—C28	1.517 (3)
O19—C19	1.402 (3)	C27—H27	1.0000
O19—C23	1.441 (3)	C28—C29	1.518 (3)
O20—C24	1.432 (3)	C28—H28	1.0000
O20—H20A	0.8400	C29—C30	1.515 (3)
O21—C26	1.422 (3)	C29—H29	1.0000
O21—H21A	0.8400	C30—H30A	0.9900
O22—C27	1.427 (3)	C30—H30B	0.9900
O22—H22A	0.8400	C31—C32	1.524 (4)
O23—C19	1.415 (3)	C31—H31	1.0000
O23—C28	1.425 (3)	C32—C33	1.519 (3)
O24—C25	1.408 (3)	C32—H32	1.0000
O24—C29	1.448 (3)	C33—C34	1.522 (3)
O25—C30	1.415 (3)	C33—H33	1.0000
O25—H25A	0.8400	C34—C35	1.526 (3)
O26—C32	1.434 (3)	C34—H34	1.0000
O26—H26A	0.8400	C35—C36	1.508 (3)
O27—C33	1.424 (3)	C35—H35	1.0000
O27—H27A	0.8400	C36—H36A	0.9900
O28—C25	1.413 (3)	C36—H36B	0.9900
O28—C34	1.435 (3)	C37—C38	1.524 (3)
O29—C31	1.408 (3)	C37—H37	1.0000
O29—C35	1.444 (3)	C38—C39	1.510 (3)
O30—C36	1.430 (3)	C38—H38	1.0000
O30—H30C	0.8400	C39—C40	1.516 (3)
O31—C38	1.422 (3)	C39—H39	1.0000
O31—H31A	0.8400	C40—C41	1.523 (3)
O32—C39	1.428 (3)	C40—H40	1.0000
O32—H32A	0.8400	C41—C42	1.510 (3)
O33—C31	1.412 (3)	C41—H41	1.0000
O33—C40	1.430 (3)	C42—H42A	0.9900
O34—C37	1.411 (3)	C42—H42B	0.9900
O34—C41	1.436 (3)	O37—H37A	0.824 (17)
O35—C42	1.432 (3)	O37—H37B	0.849 (17)
O35—H35A	0.8400	O38—H38A	0.828 (17)
O36—C12	1.410 (3)	O38—H38B	0.817 (17)
O36—H36	0.8400	O39—H39A	0.856 (18)
C1—C2	1.532 (3)	O39—H39B	0.861 (18)
C1—H1	1.0000	O40—H40A	0.879 (18)
C2—C3	1.519 (3)	O40—H40B	0.869 (18)
C2—H2	1.0000	O41—H41A	0.844 (17)
C3—C4	1.515 (3)	O41—H41B	0.840 (18)
C3—H3	1.0000	O42—H42C	0.852 (18)
C4—C5	1.527 (3)	O42—H42D	0.805 (18)
C4—H4	1.0000	O44—O44'	1.07 (3)
C5—H5	1.0000	O44—O49	1.73 (3)
C7—C8	1.539 (3)	O45—O45'	0.774 (13)
C7—H7	1.0000	O46—O46'	0.84 (4)
C8—C9	1.521 (3)	O48—O50	1.46 (4)
			
C2—O1—H1A	109.5	O23—C19—H19	109.3
C3—O2—H2A	109.5	C20—C19—H19	109.3
C37—O3—C4	117.01 (18)	O16—C20—C21	108.93 (18)
C1—O4—C5	113.34 (18)	O16—C20—C19	109.99 (18)
C6—O5—H5A	109.5	C21—C20—C19	109.15 (19)
O5—C6—C5	112.7 (2)	O16—C20—H20	109.6
O5—C6—H6A	109.1	C21—C20—H20	109.6
C5—C6—H6A	109.1	C19—C20—H20	109.6
O5—C6—H6B	109.1	O17—C21—C20	109.46 (19)
C5—C6—H6B	109.1	O17—C21—C22	110.80 (18)
H6A—C6—H6B	107.8	C20—C21—C22	110.51 (18)
C8—O6—H6C	109.5	O17—C21—H21	108.7
C9—O7—H7A	109.5	C20—C21—H21	108.7
C1—O8—C10	117.08 (18)	C22—C21—H21	108.7
C7—O9—C11	112.94 (18)	O18—C22—C23	107.67 (18)
C14—O11—H11A	109.5	O18—C22—C21	106.81 (17)
C15—O12—H12C	109.5	C23—C22—C21	112.33 (18)
C7—O13—C16	118.09 (17)	O18—C22—H22	110.0
C13—O14—C17	113.50 (17)	C23—C22—H22	110.0
C18—O15—H15A	109.5	C21—C22—H22	110.0
C20—O16—H16A	109.5	O19—C23—C24	105.91 (18)
C21—O17—H17A	109.5	O19—C23—C22	110.36 (19)
C13—O18—C22	118.62 (16)	C24—C23—C22	112.89 (19)
C19—O19—C23	113.68 (17)	O19—C23—H23	109.2
C24—O20—H20A	109.5	C24—C23—H23	109.2
C26—O21—H21A	109.5	C22—C23—H23	109.2
C27—O22—H22A	109.5	O20—C24—C23	111.1 (2)
C19—O23—C28	118.45 (17)	O20—C24—H24A	109.4
C25—O24—C29	113.37 (17)	C23—C24—H24A	109.4
C30—O25—H25A	109.5	O20—C24—H24B	109.4
C32—O26—H26A	109.5	C23—C24—H24B	109.4
C33—O27—H27A	109.5	H24A—C24—H24B	108.0
C25—O28—C34	117.86 (17)	O24—C25—O28	110.90 (19)
C31—O29—C35	113.41 (17)	O24—C25—C26	111.10 (18)
C36—O30—H30C	109.5	O28—C25—C26	108.21 (18)
C38—O31—H31A	109.5	O24—C25—H25	108.9
C39—O32—H32A	109.5	O28—C25—H25	108.9
C31—O33—C40	118.99 (16)	C26—C25—H25	108.9
C37—O34—C41	115.44 (17)	O21—C26—C27	112.28 (19)
C42—O35—H35A	109.5	O21—C26—C25	111.14 (18)
C12—O36—H36	109.5	C27—C26—C25	110.38 (19)
O4—C1—O8	111.42 (19)	O21—C26—H26	107.6
O4—C1—C2	110.68 (18)	C27—C26—H26	107.6
O8—C1—C2	107.42 (19)	C25—C26—H26	107.6
O4—C1—H1	109.1	O22—C27—C28	111.73 (19)
O8—C1—H1	109.1	O22—C27—C26	108.29 (19)
C2—C1—H1	109.1	C28—C27—C26	108.59 (19)
O1—C2—C3	112.3 (2)	O22—C27—H27	109.4
O1—C2—C1	110.80 (18)	C28—C27—H27	109.4
C3—C2—C1	110.34 (19)	C26—C27—H27	109.4
O1—C2—H2	107.7	O23—C28—C27	107.03 (18)
C3—C2—H2	107.7	O23—C28—C29	111.26 (19)
C1—C2—H2	107.7	C27—C28—C29	109.50 (19)
O2—C3—C4	106.73 (18)	O23—C28—H28	109.7
O2—C3—C2	112.39 (19)	C27—C28—H28	109.7
C4—C3—C2	110.6 (2)	C29—C28—H28	109.7
O2—C3—H3	109.0	O24—C29—C30	106.93 (19)
C4—C3—H3	109.0	O24—C29—C28	108.22 (19)
C2—C3—H3	109.0	C30—C29—C28	113.7 (2)
O3—C4—C3	107.56 (19)	O24—C29—H29	109.3
O3—C4—C5	109.00 (19)	C30—C29—H29	109.3
C3—C4—C5	110.25 (18)	C28—C29—H29	109.3
O3—C4—H4	110.0	O25—C30—C29	113.3 (2)
C3—C4—H4	110.0	O25—C30—H30A	108.9
C5—C4—H4	110.0	C29—C30—H30A	108.9
O4—C5—C6	106.7 (2)	O25—C30—H30B	108.9
O4—C5—C4	108.40 (19)	C29—C30—H30B	108.9
C6—C5—C4	114.3 (2)	H30A—C30—H30B	107.7
O4—C5—H5	109.1	O29—C31—O33	111.25 (18)
C6—C5—H5	109.1	O29—C31—C32	110.58 (19)
C4—C5—H5	109.1	O33—C31—C32	106.41 (19)
O9—C7—O13	110.19 (18)	O29—C31—H31	109.5
O9—C7—C8	111.52 (19)	O33—C31—H31	109.5
O13—C7—C8	107.64 (19)	C32—C31—H31	109.5
O9—C7—H7	109.2	O26—C32—C33	111.62 (19)
O13—C7—H7	109.2	O26—C32—C31	108.11 (19)
C8—C7—H7	109.2	C33—C32—C31	109.44 (19)
O6—C8—C9	112.0 (2)	O26—C32—H32	109.2
O6—C8—C7	109.72 (19)	C33—C32—H32	109.2
C9—C8—C7	110.97 (18)	C31—C32—H32	109.2
O6—C8—H8	108.0	O27—C33—C32	110.6 (2)
C9—C8—H8	108.0	O27—C33—C34	110.42 (19)
C7—C8—H8	108.0	C32—C33—C34	108.28 (19)
O7—C9—C8	113.35 (18)	O27—C33—H33	109.2
O7—C9—C10	110.8 (2)	C32—C33—H33	109.2
C8—C9—C10	109.68 (19)	C34—C33—H33	109.2
O7—C9—H9	107.6	O28—C34—C33	107.25 (18)
C8—C9—H9	107.6	O28—C34—C35	110.28 (19)
C10—C9—H9	107.6	C33—C34—C35	109.39 (19)
O8—C10—C9	105.82 (18)	O28—C34—H34	110.0
O8—C10—C11	111.88 (18)	C33—C34—H34	110.0
C9—C10—C11	107.94 (19)	C35—C34—H34	110.0
O8—C10—H10	110.4	O29—C35—C36	107.00 (18)
C9—C10—H10	110.4	O29—C35—C34	108.20 (19)
C11—C10—H10	110.4	C36—C35—C34	115.1 (2)
O9—C11—C12	107.95 (19)	O29—C35—H35	108.8
O9—C11—C10	106.86 (18)	C36—C35—H35	108.8
C12—C11—C10	116.8 (2)	C34—C35—H35	108.8
O9—C11—H11	108.3	O30—C36—C35	112.6 (2)
C12—C11—H11	108.3	O30—C36—H36A	109.1
C10—C11—H11	108.3	C35—C36—H36A	109.1
O36—C12—C11	115.1 (2)	O30—C36—H36B	109.1
O36—C12—H12A	108.5	C35—C36—H36B	109.1
C11—C12—H12A	108.5	H36A—C36—H36B	107.8
O36—C12—H12B	108.5	O3—C37—O34	111.60 (18)
C11—C12—H12B	108.5	O3—C37—C38	106.94 (18)
H12A—C12—H12B	107.5	O34—C37—C38	110.84 (18)
O18—C13—O14	111.31 (19)	O3—C37—H37	109.1
O18—C13—C14	107.20 (18)	O34—C37—H37	109.1
O14—C13—C14	111.02 (19)	C38—C37—H37	109.1
O18—C13—H13	109.1	O31—C38—C39	111.40 (19)
O14—C13—H13	109.1	O31—C38—C37	111.44 (18)
C14—C13—H13	109.1	C39—C38—C37	109.72 (18)
O11—C14—C15	110.39 (19)	O31—C38—H38	108.0
O11—C14—C13	109.65 (19)	C39—C38—H38	108.0
C15—C14—C13	109.79 (19)	C37—C38—H38	108.0
O11—C14—H14	109.0	O32—C39—C38	110.27 (18)
C15—C14—H14	109.0	O32—C39—C40	109.19 (18)
C13—C14—H14	109.0	C38—C39—C40	109.88 (19)
O12—C15—C16	107.38 (18)	O32—C39—H39	109.2
O12—C15—C14	110.84 (18)	C38—C39—H39	109.2
C16—C15—C14	110.75 (18)	C40—C39—H39	109.2
O12—C15—H15	109.3	O33—C40—C39	105.44 (17)
C16—C15—H15	109.3	O33—C40—C41	108.61 (17)
C14—C15—H15	109.3	C39—C40—C41	112.83 (18)
O13—C16—C15	105.71 (17)	O33—C40—H40	110.0
O13—C16—C17	110.33 (18)	C39—C40—H40	110.0
C15—C16—C17	112.08 (19)	C41—C40—H40	110.0
O13—C16—H16	109.5	O34—C41—C42	105.01 (18)
C15—C16—H16	109.5	O34—C41—C40	111.49 (18)
C17—C16—H16	109.5	C42—C41—C40	111.44 (19)
O14—C17—C18	105.75 (18)	O34—C41—H41	109.6
O14—C17—C16	109.75 (18)	C42—C41—H41	109.6
C18—C17—C16	113.1 (2)	C40—C41—H41	109.6
O14—C17—H17	109.4	O35—C42—C41	111.42 (18)
C18—C17—H17	109.4	O35—C42—H42A	109.3
C16—C17—H17	109.4	C41—C42—H42A	109.3
O15—C18—C17	107.79 (19)	O35—C42—H42B	109.3
O15—C18—H18A	110.1	C41—C42—H42B	109.3
C17—C18—H18A	110.1	H42A—C42—H42B	108.0
O15—C18—H18B	110.1	H37A—O37—H37B	105 (2)
C17—C18—H18B	110.1	H38A—O38—H38B	113 (3)
H18A—C18—H18B	108.5	H39A—O39—H39B	103 (3)
O19—C19—O23	110.45 (19)	H40A—O40—H40B	95 (2)
O19—C19—C20	110.13 (18)	H41A—O41—H41B	109 (3)
O23—C19—C20	108.29 (18)	H42C—O42—H42D	111 (3)
O19—C19—H19	109.3	O44'—O44—O49	139.5 (19)

### Hydrogen-bond geometry (Å, °)

**Table:**

*D*—H···*A*	*D*—H	H···*A*	*D*···*A*	*D*—H···*A*
O1—H1A···O7	0.84	2.36	3.065 (3)	142
O2—H2A···O45^i^	0.84	2.00	2.809 (3)	161
O2—H2A···O45'^i^	0.84	1.95	2.633 (11)	138
O5—H5A···O47	0.84	1.76	2.578 (15)	163
O5—H5A···O45^ii^	0.84	2.46	3.024 (4)	125
O5'—H5'···O45^ii^	0.84	1.66	2.47 (3)	163
O5'—H5'···O45'^ii^	0.84	2.40	3.20 (3)	159
O6—H6C···O12	0.84	2.04	2.845 (2)	161
O6—H6C···O13	0.84	2.31	2.742 (2)	112
O7—H7A···O26^i^	0.84	2.14	2.956 (3)	165
O11—H11A···O40	0.84	1.78	2.595 (2)	163
O12—H12C···O31^iii^	0.84	1.85	2.676 (2)	167
O15—H15A···O41^iv^	0.84	2.04	2.870 (3)	172
O16—H16A···O38^v^	0.84	1.90	2.733 (3)	174
O17—H17A···O11	0.84	2.06	2.889 (2)	171
O20—H20A···O43	0.84	1.93	2.725 (3)	157
O20—H20A···O50	0.84	2.12	2.80 (3)	138
O21—H21A···O27	0.84	1.99	2.810 (2)	167
O21—H21A···O28	0.84	2.36	2.794 (2)	112
O22—H22A···O16	0.84	1.97	2.776 (2)	160
O22—H22A···O23	0.84	2.41	2.821 (2)	111
O25—H25A···O39^vi^	0.84	1.90	2.743 (3)	178
O26—H26A···O37^iii^	0.84	1.83	2.668 (3)	176
O27—H27A···O5^v^	0.84	2.09	2.821 (3)	145
O30—H30C···O15^vii^	0.84	2.06	2.858 (3)	158
O30—H30C···O14^vii^	0.84	2.37	2.945 (2)	126
O31—H31A···O2	0.84	2.01	2.844 (2)	172
O32—H32A···O26	0.84	2.04	2.871 (2)	170
O35—H35A···O37^i^	0.84	2.08	2.890 (2)	161
O36—H36···O11^ii^	0.84	2.02	2.854 (2)	173
O37—H37A···O6	0.82 (2)	2.05 (2)	2.837 (2)	160 (3)
O37—H37B···O32^viii^	0.85 (2)	1.91 (2)	2.753 (2)	171 (3)
O38—H38A···O20	0.83 (2)	2.11 (2)	2.865 (3)	152 (3)
O38—H38B···O30^ix^	0.82 (2)	1.86 (2)	2.672 (3)	176 (3)
O39—H39A···O22^ii^	0.86 (2)	1.90 (2)	2.749 (3)	173 (3)
O39—H39B···O38	0.86 (2)	2.18 (2)	2.959 (3)	151 (3)
O40—H40A···O41^x^	0.88 (2)	2.21 (3)	2.787 (3)	123 (3)
O40—H40B···O35^xi^	0.87 (2)	1.91 (2)	2.770 (3)	168 (3)
O41—H41A···O20	0.84 (2)	2.13 (2)	2.976 (3)	175 (3)
O41—H41A···O19	0.84 (2)	2.63 (3)	3.048 (2)	112 (3)
O41—H41B···O25	0.84 (2)	1.95 (2)	2.769 (3)	166 (3)
O42—H42C···O21^ii^	0.85 (2)	2.09 (2)	2.932 (3)	173 (3)
O42—H42D···O48	0.81 (2)	1.90 (4)	2.53 (3)	135 (3)
O42—H42D···O39	0.81 (2)	2.34 (3)	3.038 (3)	146 (3)

Symmetry codes: (i) −*x*+1, *y*+1/2, −*z*; (ii) *x*, *y*+1, *z*; (iii) −*x*+1, *y*−1/2, −*z*; (iv) −*x*+2, *y*+1/2, −*z*+1; (v) *x*, *y*−1, *z*; (vi) −*x*+2, *y*−1/2, −*z*; (vii) *x*, *y*, *z*−1; (viii) *x*, *y*, *z*+1; (ix) −*x*+2, *y*+1/2, −*z*; (x) −*x*+2, *y*−1/2, −*z*+1; (xi) *x*, *y*−1, *z*+1.
